# Trace elements geochemistry in high-incidence areas of liver-related diseases, northwestern Ethiopia

**DOI:** 10.1007/s10653-019-00387-3

**Published:** 2019-09-10

**Authors:** Jemal Ahmed

**Affiliations:** Department of Earth Science, University of Samara, Samara, Ethiopia

**Keywords:** Trace elements geochemistry, Liver disease, Environmental health, Maximum acceptable concentration (MAC), Tigray, Ethiopia

## Abstract

This paper reports the results of trace elements geochemistry from Tigray national state, northwestern Ethiopia. The area is part of the Arabian-Nubian Shield, where the dominant exposure is low-grade metamorphic rocks and has a long history of liver-related diseases. The increase in the number of liver-related disease patients of the area has been an environmental health issue of national concern. The aim of the study is to determine the level of trace element concentrations and distributions in water and stream sediments of the area and identify the possible sources in relation to human health. Water, stream sediment and rocks samples (20 water, 20 stream sediments, and 6 rock samples) were collected in March 2011 and analyzed for major and trace element contents using ICP-MS, ICP-OES, ion Chromatography, and XRF methods. Bromine, aluminum, fluorine, arsenic, and nitrate values exceed the WHO maximum acceptable concentration (MAC) for drinking purpose. Bromine ranges from 0.11 to 1.48 mg/l show higher values in all samples, and fluorine ranges from 0.21 to 16.49 mg/l show higher values in 20% of the samples. Other trace elements are aluminum—30%, arsenic—10%, and nitrate (NO_3_)—10%, and they are examples of elements which have above MAC for drinking water. Selenium deficiency may be the other problematic element in the area for which its deficiency is associated with liver damage and heart muscle disorder. The concentration of cobalt and chromium exceeded world geochemical background value in average shale at most sample stations indicated that these stations were in potential risk.

## Introduction

**Fig. 1:**
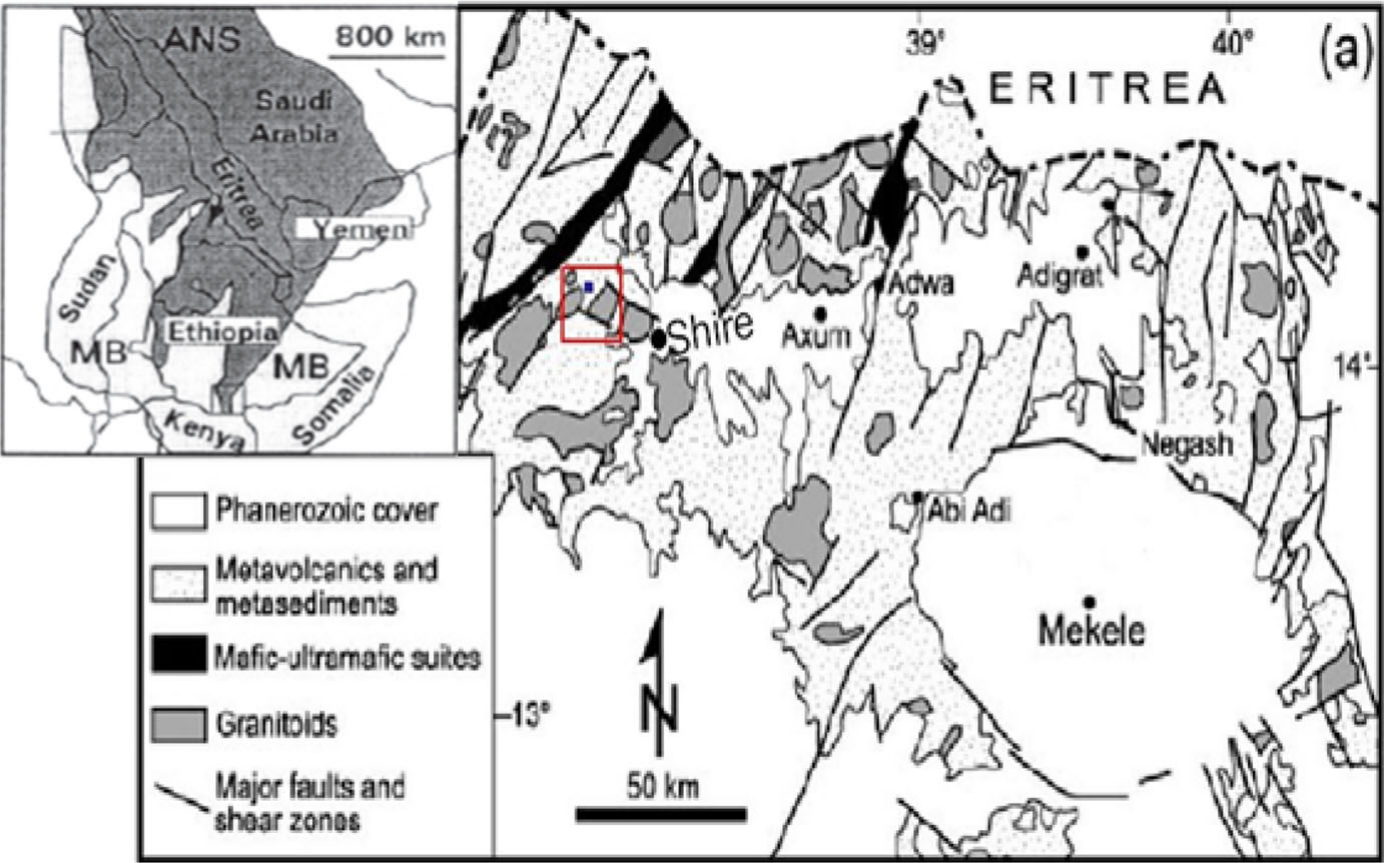
Geological maps of northern Ethiopia (Asrat 2001)

The Shire area is part of the Arabian-Nubian Shield which extends from Saudi Arabia and Egypt (Fig. 1) in the north, and dawn to Ethiopia is believed to have developed by Phanerozoic-type plate—tectonic process during the 950–500 Ma, Pan-African orogeny (Stern 1994).

There are a number of diseases proved to be connected to geochemical characteristics of the environment (Fergusson 1990). Well-known examples are: The endemic degenerative heart disease in China known as Keshan disease was attributed due to selenium deficiency (Yang and Xia 1995) and kidney disease in Sri Lanka, whereas high-fluoride content in drinking water was considered as possible risk factors (Dissanayake 1991). Arsenic poisoning in Bangladesh which is caused by high arsenic concentration in the aquifer is the most serious arsenic problem in the world. Studies of the causative–consecutive relations between geochemistry and human health are very complex and subject to many investigations (Little 2004).

It had been reported that people in Shire area were dying from a new disease characterized by “Gua Kua” (stomach swollen). Interviews with the local people indicated that the liver-related diseases were identified in the area since 1980; about 70 peoples or more have been died so far. Males and females have been equally affected, and children aged between 7 and 15 appear to be most susceptible. With time, the rate of disease is increased and affected more people (Tigray Health Bureau 2009).

In 2006, the Tigray Regional Health Bureau sent national task forces from Ethiopian Health Nutrition and Research Institute (EHNRI) and Addis Ababa University (Tikur Anbessa Hospital) into the area to investigate the cause of this disease. They took plant samples and it was analyzed by national EHNRI laboratory. The result interpreted as a potential toxic plant called “Ageratum” growing around the water source. The “Ageratum” contained a pyrrolizidine alkaloid and that the “Gua Kua” disease was a veno- occlusive disease (VOD) of the liver. As a result of this report, the water supply in Tsada Emba—northeastern Shire—was filled in and the people internally displaced into the resettlement area in Kelakil—southeastern Shire (Tigray Health Bureau 2009).

No reduction in incidence was noted after the resettlement program. The Tigray Health Bureau was forced to question the diagnosis, which led in turn in 2008 to a request to United State Center for Disease Control (CDC) to investigate the crisis in the area. They conducted a case control study and took serum samples and concluded their report with schistosomiasis as causative agents (Oasis Foundation of Ethiopia 2009).

Oasis Foundation of Ethiopia (OFE) made contact with Imperial College of London who runs the Schistosomiasis Control Initiative (SCI). SCI sent a team to investigate a schistosomiasis and took (nail, stool, and urine) samples and were able to conclude convincingly schistosomiasis was not a cause of morbidity (Fergusson 1990). Reports from EHNRI, CDC, and SCI were reviewed by professor Thursz from Imperial Collage of London and agreed with the EHNRI reports that the most likely cause would be a pyrrolizidine alkaloid as originally described with bush tea disease in Jamaica. However, it seemed unlikely that either “Ageratum” or the water supply could be a pyrrolizidine alkaloid (Oasis Foundation of Ethiopia 2009).

**Fig. 2:**
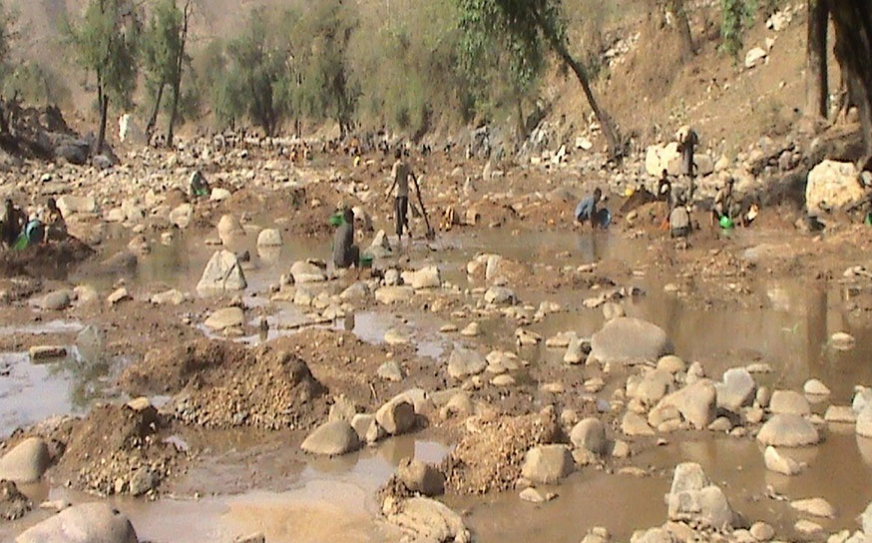
Artisanal gold mining practices in the area (southeastern view)

In the study area, there are abandoned and ongoing artisanal gold mining sites (Fig. 2). Many individuals have been panning for gold using manual operations for a long time, and there are many intermittent streams in the area into which drainage from gold mining sites flows into main rivers, and this may have led many people to ask whether the illnesses in the community have an environmental cause.

For the protection and conservation of water resource in the study area, it is found to be important to characterize the quality of water. Groundwater contamination with arsenic, fluoride, and nitrate recently possesses serious health hazards to large sector of communities all over the word (Dissanayake 1991). Since sediments and suspended particles are important repositories for trace metals such as chromium, copper, molybdenum, cobalt, and manganese, studying of sediments of the environment is also found to be vital for characterization of the quality of the environment of the artisanal gold mining sites in and around the study area.

Trace element concentrations in the natural water vary widely depending on the geochemistry of rocks in the immediate environment. Interactions of water and plants with rocks (and soils developed from them) dictate our intake of these elements (Santra 2001). So, knowledge of rock types in a particular area can help find out potential health problems that may be associated with concentration of particular elements.

### General overview of the study area

#### Location and population size

**Fig. 3:**
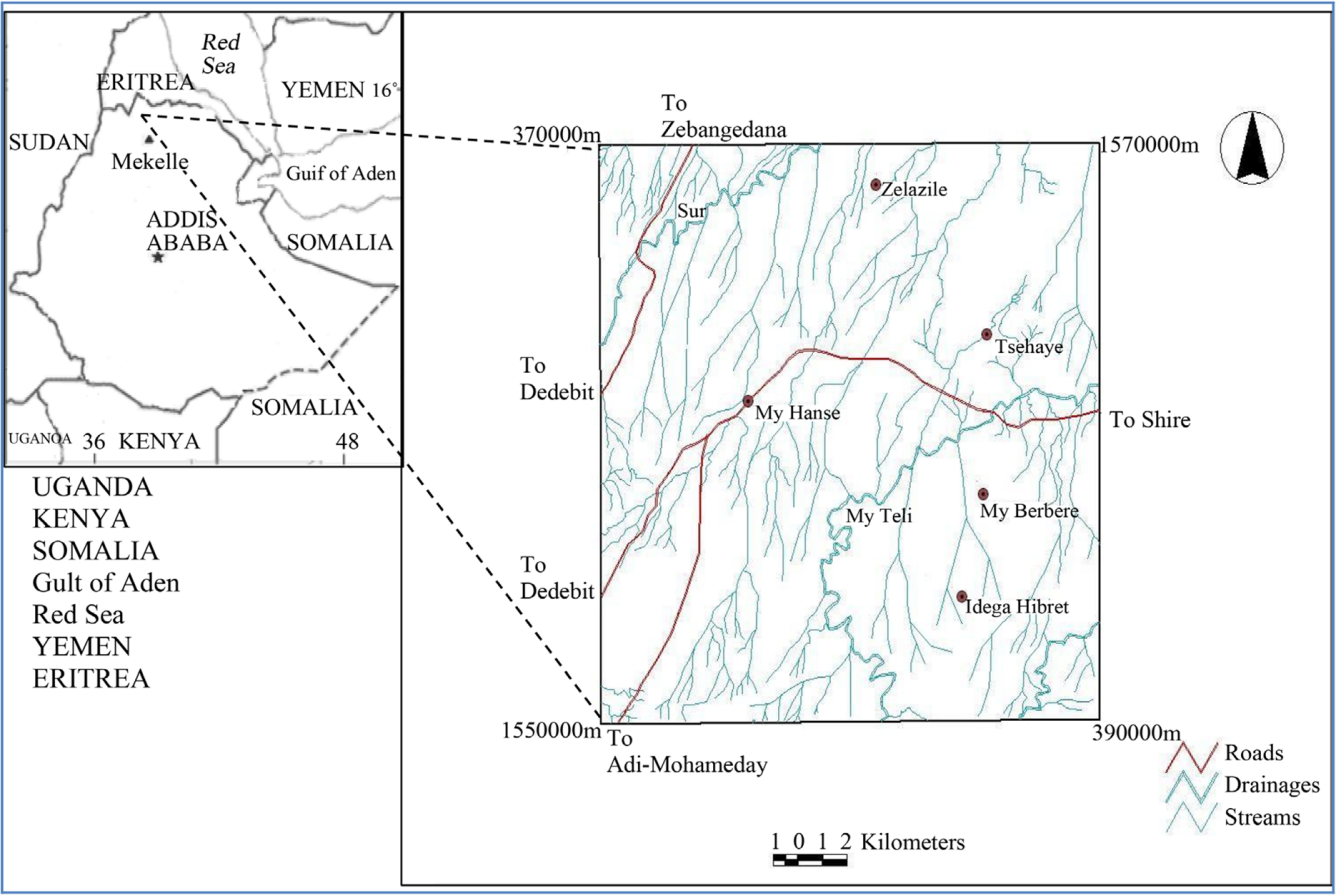
Location map of Asgede Tsimbla sub-catchments area

The study area is located about 355 km northwest of Mekelle, Tigray national state, northern Ethiopia. Geographically it is located 1,550,000–1,570,000 m (14°01′12″ to 14°11′54″) north latitudes and 370,000–3,90,000 m (37°47′48″ to 37°58′54″) east longitudes (Fig. 3). Access with in the study area is possible through weathered road that connects Shire town to the study area. In general, it can be said that the area is scarcely populated and population density varies from place to place. The population densities of the area are around 25,143, and from this 12,103 are men and 13,040 are women according to Central Statistical Agency of Ethiopia (CSA) (2005).

#### Climate and topography

It is characterized by semiarid to arid climate. The average annual temperature for the region in generally varies from 24 to 29 °C. Records obtained show temperature maxima of between 37 and 40 °C and minima of 15–19 °C. November and December are the coldest months (National Meteorological Service Agency (NMSA) 2008). It has an overall topography that decreases from northeast toward southwest. Metavolcanic and metagranite generally occupy the high ground while the metasediments predominantly occupy the river valleys. The altitude ranges from 1340 to 860 meters above sea level. Streams are intermittent and often drain southwest direction. The drainage pattern is sub-dendritic to well-developed dendritics. The main streams in the study area are: Mai-Hammar, Mai-Teli, Mai-Weyle, Sembel, and Fesfesay rivers.

#### Geological settings

**Fig. 4:**
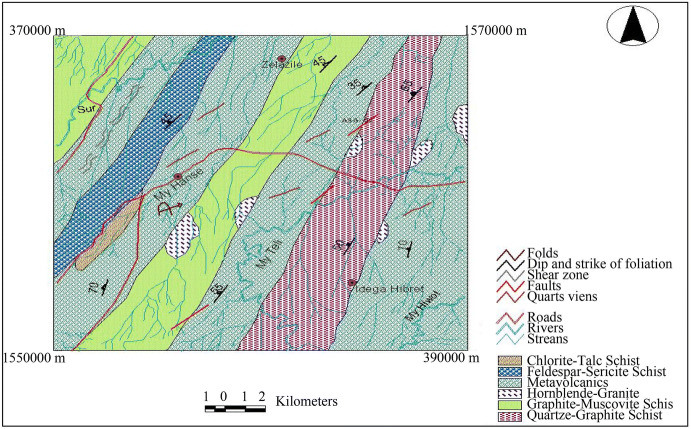
Geological map of Asgede Tsimbla sub-catchment area

The area of interest (Mai-Hanse) and its surroundings are covered by metamorphic rocks which include metasediments represented by slate, graphite–muscovite schist and quartz–graphite schist in its central, eastern, and western parts, and intermediate metavolcanics exposed in the eastern, northwestern, and central, which in turn are underlain by mafic–ultramafic belt and intruded by a circular granitic bodies exposed at the south and northeastern part of the prospect area. Aplitic dykes and quartz vein and veinlets are also exposed in different parts of the area cutting the basement rocks (Fig. 4). The lithology of the area is affected by different structures such as folds, faults, fractures, and shear zones. The other major structural feature in the area is that northeast–southwest striking and northwest dipping composite foliations.

## Methodology

### Data collections

Collection and review of previous data on the area were preformed. Literature review focused on the geology, geological structures, hydrogeology, and environmental situations of the area. Topographic map at 1:50,000 scale was used to illustrate the drainage and physiographic of the area. Preliminary field survey was conducted just before the actual sampling survey by our supervisors.

**Fig. 5:**
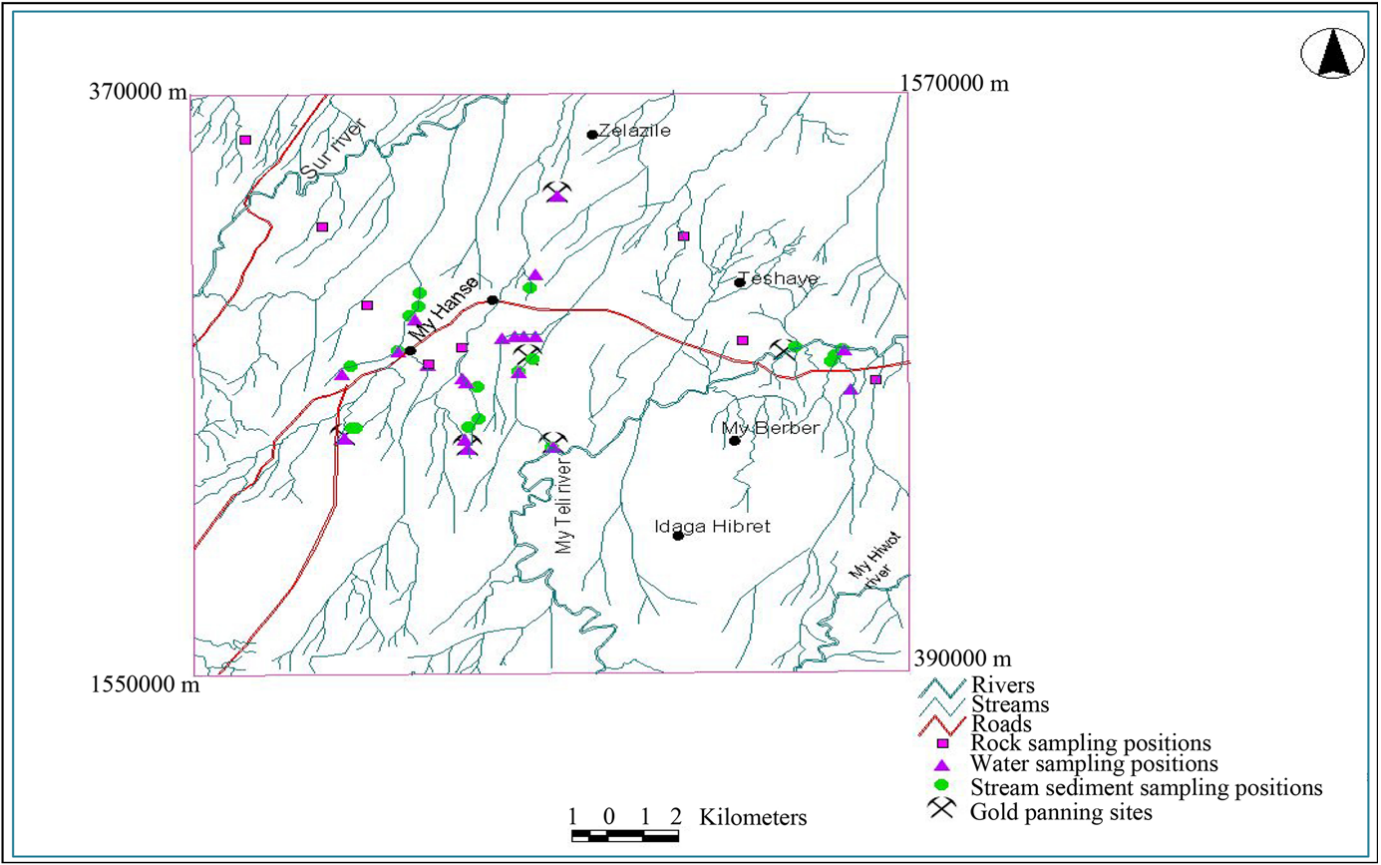
Location map of study area with sample locations and gold panning sites

The sampling sites of water and stream sediments were selected based on the distribution of potential pollutant sources. In conjunction with sampling, close field observations were made on the types of geology, physical land degradation, and traditional gold panning practice. Our sampling positions and supplementary information are presented in Fig. 5.

#### Water sampling

Thirteen (13) groundwater and seven (7) surface water samples were taken from deep borehole, shallow hand-dug wells, rivers, and ponds. Each sample was collected in one-liter polyethylene bottle, and the sampling bottle was rinsed repeatedly with groundwater before taking the samples.

After sampling, the bottles were tightly covered with caps and sealed with tap to minimize oxygen contamination and the escape of dissolved gases. The samples are kept in cool place to minimize chance of chemical reaction which can result in precipitation of dissolved elements. Information about the sampling sites (i.e., depth of the wells, how far the wells are from the artisanal mining sites and possible major contaminants) was also collected when possible in the field.

#### Stream sediment sampling

Twenty (20) stream sediment samples were collected from dry and wet stream sediments. Since some tributaries of seasonal streams have had no water flow for many months, the stream bed was covered by fallen bank materials. The fallen bank material has been removed by digging, and the stream sediment was sampled with much care. Most of stream sediment samples were taken from the same site where water sample is collected. About 300 g the samples were collected using a shovel tool and store into a clean plastic bags.

#### Rock samples

Representative rock samples were collected from all mappable units, and six of them are selected for thin section and XRF analysis. The lithology, structures, mineralization, quartz veins, and alteration intensity of the rocks have been studied in the field. A generalized geological map at 1:50,000 scale is prepared based on the satellite image and field data (Fig. 4).

### Sample analyses

The chemical analysis of water samples was carried out in the Institute for Applied Geosciences Laboratory at Graz University of Technology, Austria.

#### Water analysis

Trace elements (Cd, As, Se, Pb, Cr, Ni, Cu, Co V, Sr, Tm, and Ba) were analyzed using inductively coupled plasma mass spectrometer (ICP-MS; Perkin-Elmer Elan 5000) with ultrasonic nebulization, and it was calibrated with multi-element standard solutions. The detection limit ranges between 0.01 and 0.1 µg/l, and precision also reported as 10% for numerous trace elements. Major cations (Ca^+2^, Mg^+2^, Na^+^, and K^+^) were analyzed using inductively coupled plasma optical emission spectrometry (ICP-OES, Perkin-Elmer 4300) and anions (Cl^−^, NO_3_^−^, and SO4^−2^) were measured using ion chromatography (IC; Dionex 600)/HPLC high-performance liquid chromatography.

#### Rock and stream sediment analyses

Rock samples were first crushed in stainless steel jaw crusher and then powdered in an agate mill for chemical analysis, and the sample preparation for stream sediments also involves drying in an oven, crushing, grinding, sieving with −200 mesh, and packing about 100 mg with plastic bags in Ezana Mining Laboratory, Mekelle. Both rock and stream sediment samples have been sent for trace elements (Cd, As, Se, Pb, Cr, Ni, Cu, Co V, Sr, Tm, Hg, Ba, Zn, Mn, and Mo) and major oxides (SiO_2_, Al_2_O_3_, Fe_2_O_3_, LOI, and MgO) analysis using X-ray fluorescence (Philips PW 2404) spectrometer at the laboratory of Institute for Geoscience, Graz University of Technology, Austria. It was done by two methods—pressed powder pellet for trace elements and fused glass for major oxides. The samples were initially ground and dried at 110 °C (in fused glass) and 60 °C (pressed powder pellet) overnight to remove remaining moisture.

Fused glass disks were created in such a way that constituent powder of 1 g was mixed homogeneously with 6 g dilithium tetraborat. This mixture was melted at 1030 °C for few minutes and poured into Pt–Au crucible and used to be run for major oxide analyses. The techniques used for fused glass are a modification of the methods described by Thomas and Haukka (1978). Trace elements were determined using pressed powder pellets, where the pellets were made by mixing of rock powder of 12 g with pulverizer wax of 3 g and pressing the mixture under a maximum pressure of 400 bars to make a pressed pellet of the sample. This wax-pressed tablet requires filling up a small aluminum dish with finely powdered sample mixed with glue, in order to have the individual grains stick together. The homogenized mixture was compressed, and once the pressure is released, it solidifies into a compact disk with a smooth upper surface that can easily be measured in the XRF spectrometer. Accuracy and precision for XRF data are the same as those given in Reimold et al. (1994).

### Treatments of analytical data

Analytical treatment of data was made in Excel, SPSS, Aquachem, and Archview. When analyzing in Aquachem, SPSS, and Archview, the value below detection limit level was set to zero. Statistical analysis such as mean, median, standard deviation, and multivariate analysis (correlation) was used for data analysis.

Data obtained from water samples were compared with the national and international standards (World Health Organization (WHO) 2004; Ethiopian 2001; US EPA 2001) to see whether or not the water samples are in line with the recommended range, and they are safe to human health. The analytical results of stream sediments are compared with world geochemical background value in average shale (Turkian and Wedpohl 1961).

## Results

### Lithogeochemistry

Major and trace element analysis of some rock samples obtained from the study area was carried out to find out potential health problems associated with concentrations of particular elements (Table 1).

**Table 1:** Major (wt%) and trace (ppm) element compositions of selected rock samples in the study area

Rock type	Graphite schist (ATR-3)	Graphite–muscovite schist (ATR5)	Feldspar–Sericite schist (ATR1)	Metavolcanics (ATR2)
*Major oxides*				
SiO_2_	70.39	64.17	64.21	67.27
Al_2_O_3_	15.11	18.55	15.99	15.58
Fe_2_O_3_	2.72	5.69	6.85	6.04
LiO	4.96	5.89	2.43	1.57
Na_2_O	1.44	1.06	4.43	3.67
MgO	1.15	1.5	1.59	1.84
K_2_O	2.62	2.15	1.69	1.27
TiO	6.65	0.69	1.63	0.68
Mn	0.11	0.16	0.08	0.05
P_2_O_5_	0.02	5.16	0.19	0.2
CaO	0.83	0.1	0.88	1.82
Total	106	105.08	99.97	99.99
*Trace elements*				
Cu	28	153.1	67.5	14.8
Cr	222.3	147.6	149.5	67.9
Mn	786	1342.8	536.8	708.5
Sr	111.1	34.6	267	162.3
Zr	195	274.1	162.5	157.6
Ba	182.6	1939	384.5	598.8
Rb	73.3	67.2	41.7	37.6
pb	11.7	12.3	9.4	5.5
Ni	17.7	54.7	18.4	9
Zn	51.7	107.2	62.4	71.6
Co	1.3	14.3	10.5	7.9
Cd	4.4	5	5.8	5.3
Hg	bdl	bdl	bdl	bdl
Sb	bdl	bdl	bdl	bdl
Cs	bdl	bdl	bdl	bdl
Ce	29.4	55.1	27.7	29.7
U	3.3	4.1	0.5	0.1
V	193.4	162.7	89.4	88.3

### Hydrogeochemistry

The analytical results and summary statistics (mean, minimum, maximum, and standard deviation) of selected elements in water samples from Asgede Tsimbla sub-catchment area are presented in Table 2. It gives the analytical techniques for different elements/parameters and shows how many orders of magnitude the natural concentration of the analyzed elements cover in this data set. It provides additional information on water standards (World Health Organization (WHO) 2004; Ethiopian 2001; US EPA 2001) and shows the percentage of samples above maximum acceptable concentrations (MAC) limits.

**Table 2:** Range, mean and standard deviation of the analytical data for selected elements in water samples and their comparison with different water standards

Parameters	Techniques	Units	Range	Mean	SD	Standards (MAC)			> MAC* (%)
						WHO (2004)	Ethiopian (2001)	US EPA (2001)	
As	ICP-MS	µg/l	bdl–23.7	2.47	6.73	10	10	10	10
Pb	ICP-MS	µg/l	bdl–24.6	1.24	5.34	10	20	10	5
Al	ICP-MS	µg/l	bdl–553	73.75	137.76	200	400	50–200	30
Fe	ICP-MS	µg/l	bdl–1939	1.49	3.69	300	400	300	20
Cd	ICP-MS	µg/l	bdl–0.21	0.068	0.048	3	3	3	–
Tm	ICP-MS	µg/l	bdl–0.40	0.031	0.068	–	–	2	–
Ni	ICP-MS	µg/l	bdl–0.255	0.04	0.06	20		–	–
Co	ICP-MS	µg/l	bdl–0.56	0.122	0.138	–	–	–	–
Se	ICP-MS	µg/l	bdl–13.99	2.16	3.04	10	10	10	5
U	ICP-MS	µg/l	0.05–4.32	0.88	1.10	2	–	–	5
Br	IC	mg/l	0.11–1.48	0.67	0.405	0.01	–	0.01	100
F	IC	mg/l	0.21–16.5	1.86	4.12	1.5	3.0	4.0	20
NO_3_	IC	mg/l	0.01–51.8	11.93	1.75	–	50	10	10
SO_4_	IC	mg/l	1.12–435	69.5	8.1582E1	500	–	–	–
Mg	ICP-AES	mg/l	7.56–127	59.68	3.3952E1	–	–	–	–
pH			6.11–8.3	7.12	0.653	–	6.5–8.5	6.5–8.5	

> MAC* (%) = greater than maximum acceptable concentration for number of samples in percentage

#### Major ions

The collected surface and groundwater samples were analyzed for major cations (Ca^+2^, Mg^+2^, Na^+^, and K^+^) and anions (Cl^−^, NO_3_^−^, and SO4^−2^), and only two major ions (i.e., Mg^+2^ and NO_3_^−)^ were exceeded the quality standards set for drinking water (Table 2). In terms of chemical composition generally it classifies as: About 60% of the samples have Mg and the rest has Ca followed by Na as dominating cations; according to anion NO_3_ as dominate followed by Cl and SO_4_. The water composition can also be arranged as Mg^2+^ + Ca^2+^ > Na^+^ + K^+^ and SO_4_^2−^ + Cl^−^ > NO_3_^−^ +CO_3_^2−^.

#### Trace elements

The nature and spatial distribution of trace element concentration in surface water and groundwater of the area found as geochemical indicator of liver cancer. Out of 35 trace element analyses, the following seven elements show value exceeding the maximum acceptable concentration (MAC) limits (Table 2): Br, Al, Fe, F, As, Pb, and U according to WHO standards (World Health Organization (WHO) 2004).

##### Bromine

**Fig. 6:**
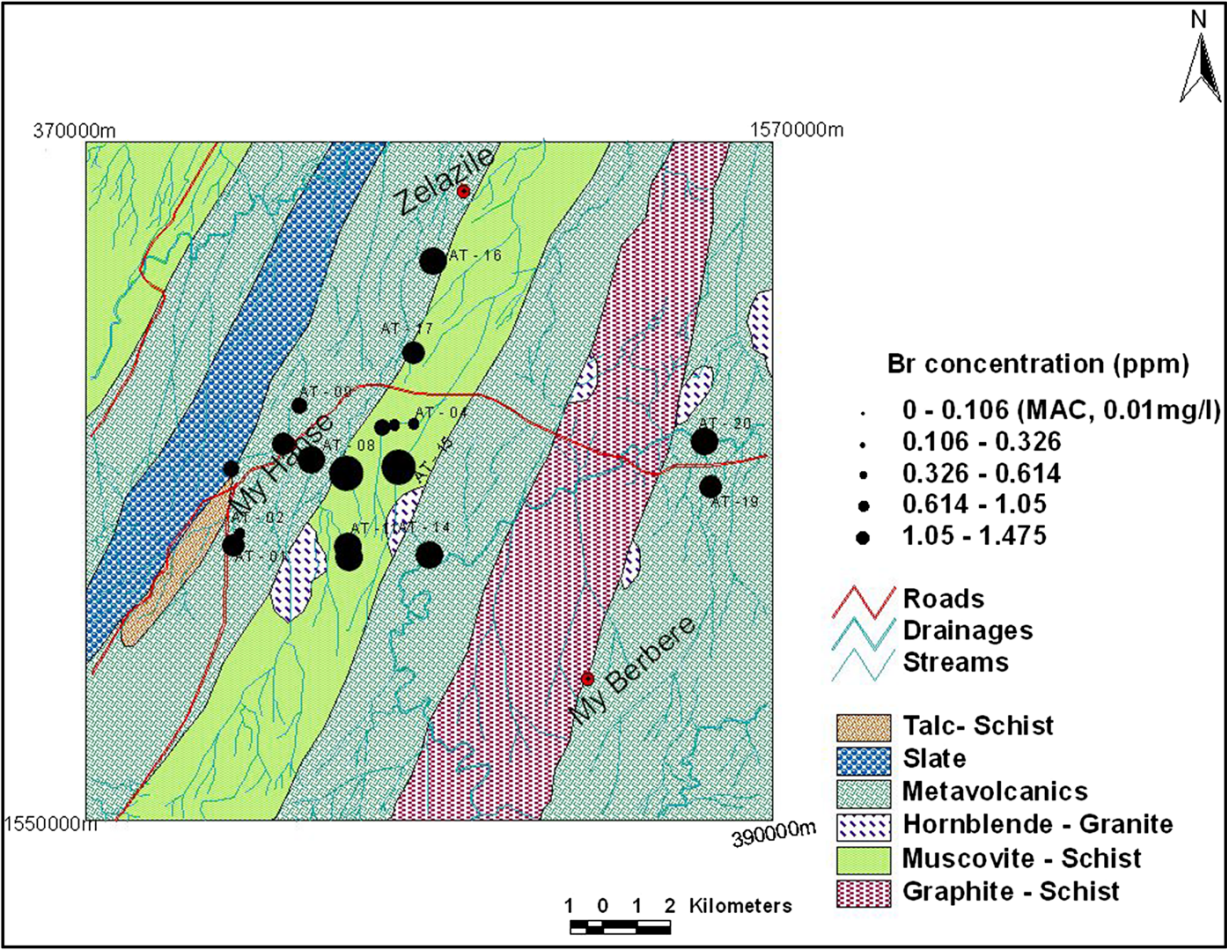
Spatial distribution of bromine concentration

The results of bromine range from 0.11 to 1.48 mg/l with mean value of 0.67 mg/l. In the study area, 1.48 mg/l is maximum in the groundwater, whereas 0.93 mg/l has to be in the surface water. The highest bromine value (AT-15) is found in the borehole around Mai Lomine Village. All the results are found to be above WHO maximum allowable level—0.01 mg/l—(Table 2). The spatial distribution of fluorine is shown in Fig. 6.

##### Fluorine

**Fig. 7:**
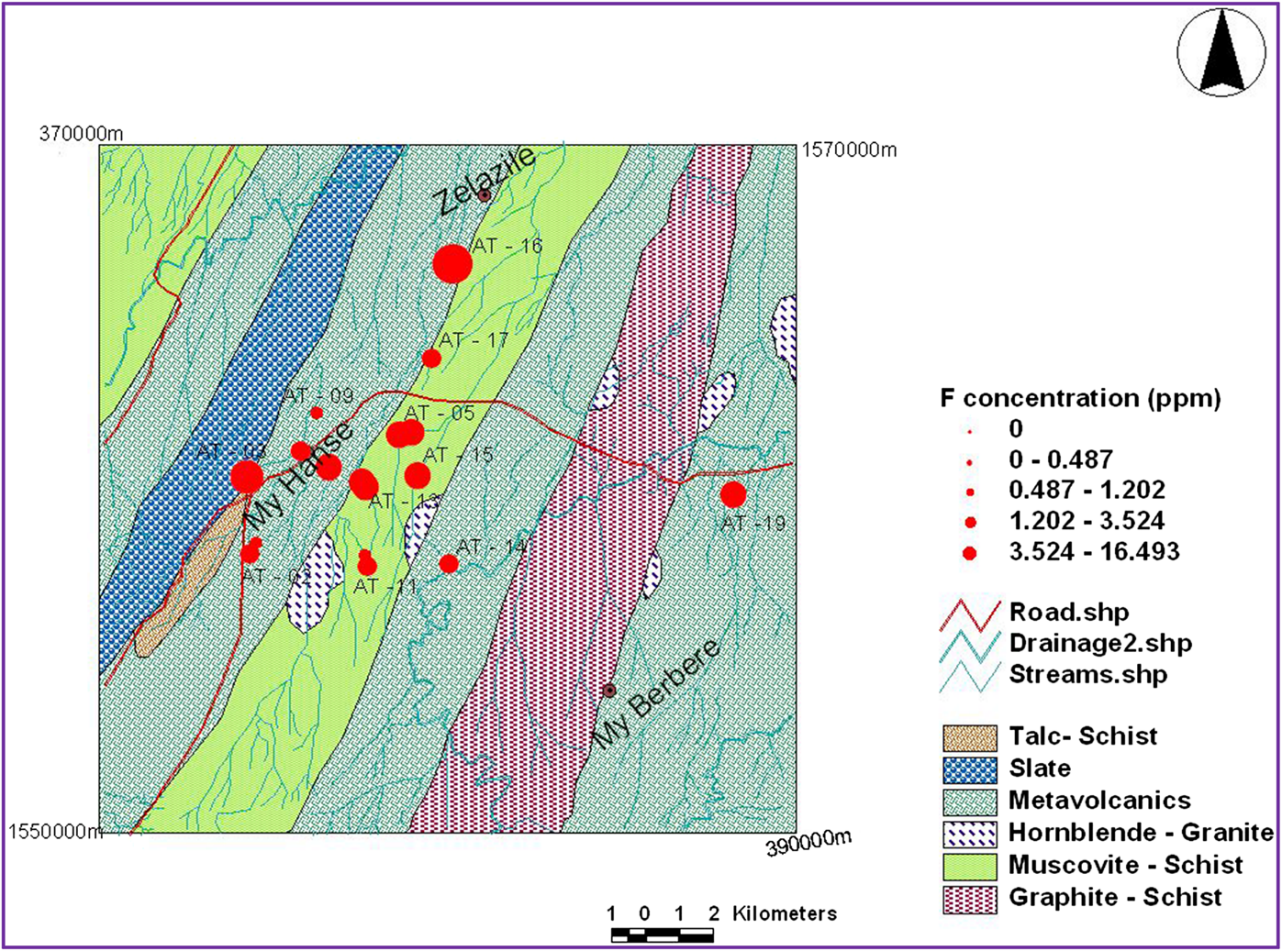
Spatial distribution of fluorine concentrations

The surface and groundwater collected from various sites of Asgede Tsimbla area contained fluorine of 0.21–16.49 mg/l with an average value of 1.18 mg/l (Table 3). The maximum value of 16.49 mg/l recorded in sample (AT-16) (Fig. 7) collected from borehole at Sembel Village, NE of Mai-Hanse Town, while the maximum value of 0.79 mg/l collected from surface water. Four of the samples indicate values higher than maximum allowable limit (1.5 mg/l) of WHO (2004) drinking water guideline (Table 3). The spatial distribution of fluorine is shown in Fig. 7.

**Table 3:** Analysis results of heavy metals in stream sediments (ppm) and its mean value in comparison with world geochemical background value in average shale

Sample no.	Zn	Ni	Pb	Cu	Co	Ag	Cr	Mn
ASG-01	70	33	1	42	39	1.4	77.8	539
ASG-02	79	65	9	71	52	1.5	68.9	708.
ASG-03	59	24	5	29	30	1.1	131	359
ASG-04	63	37	5	38	34	1.2	227	451
ASG-05	63	44	4	45	37	1.3	154	1100
ASG-06	71	34	3	45	30	1.2	188	410
ASG-07	105	37	6	44	29	1.2	171	399
ASG-08	66	29	4	32	22	1.9	172	265
ASG-09	33	18	3	20	24	2.0	160	536
ASG-10	64	28	3	30	19	1.76	149	255
ASG-11	76	32	8	38	21	2.2	147.6	354
ASG-12	68	29	8	34	24	2.1	151.2	231
ASG-13	57	26	5	30	20	1.6	143	350
ASG-14	78	36	6	43	25	1.8	128.4	255
ASG-15	50	22	7	29	18	2.3	164.6	354
ASG-16	107	54	12.3	37	19	2.2	132.4	229
ASG-17	84.7	41	11.4	31.6	32	2.4	136.2	260
ASG-18	86	45	13.6	33	37	2.7	147.6	786
ATS-19	26.3	18	16.5	11	48	5.2	251.3	623
ATS-20	48.9	19	6.5	75	54	5.3	160.6	478
Minimum	26.3	18	1	11	18	1.1	68.9	229
Maximum	107	65	16	75	54	5.3	251	1100
Mean	67.7	33.55	6.86	37.8	30	2.1	152.8	447.1
Average shale**ª**	95	68	20	45	19	–	90	850

Average shaleª: world geochemical background value in average shale (Turkian and Wedpohl 1961)

##### Aluminum

The surface and groundwater collected from various sites of Asgede Tsimbla area contained Al (filtered concentration) from less than the detection limit (0.01 ppb) up to 553 ppb, with an average value of 73.75 ppb (Table 2). The highest concentration of Al was observed in a sample (AT-07) collected from borehole near Mai-Hanse Town while in surface water 155 ppb was observed as a maximum value. 30% of the sample value indicates above MAC (i.e., 100 µg/l)-based Canadian guideline for drinking water (1996).

##### Arsenic

The concentration of arsenic ranges from less than the detection limit (0.01 µg/l) up to 23.7 µg/l with a mean value of 2.47 µg/l (Table 1). 23.7 µg/l is maximum concentration in groundwater while 20.2 µg/l in the surface water. The highest values were recorded in samples (AT-10 and AT-11) collected around Fesfesay River, where intensive artisanal gold mining had been taking place and is located south–southeast of Mai-Hanse Town. Only two samples (10%) fall above MAC of As (10 µg/l) in the study area.

##### Selenium

The value of Se varied from less than detection limit (0.01 ppb)—13.9 ppb with an average value of 2.16 µg L^−1^. In the study area, the maximum value in ground and surface water was recorded as 13.9 ppb and 4.70 ppb, respectively, and only one sample (AT-20) exceeds the WHO safe limits. The maximum value of 13.9 ppb (AT-20) was observed in a sample collected from Mai-Hammar River, where artisanal gold mining practices had been carried out.

#### Bivariate correlations

**Fig. 8:**
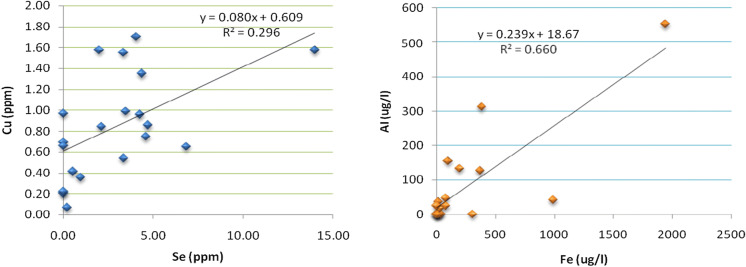
Diagram showing correlation for Cu and Se and for Al and Fe

The correlations of selected elements, where some of them fall above MAC values, are presented in “Appendix 4.” To visualize the correlations, scatter plots (which determine potential relationship among these elements) are constructed for some of these elements (Fig. 8).

### Stream sediment geochemistry

The analytical results and summery statistics value of stream sediments are listed in Table 3). It gives additional information on world geochemical background value in average shale of each element concentration.

## Discussion

### Lithogeochemistry

The composition of surface water and shallow groundwater will closely reflect the local geology. Weathering acting on mineral deposits contributes to promote a local rise of the contents of major, minor, and trace elements. These anomalous can be identified in soil, stream sediment, and waters (Komatina 2001).

In terms of trace and major element analysis, the metavolcanics, feldspar–sericite schist and graphite schist samples are enriched with Mo, Cu, Mn, Cr, and Rb elements and have high SiO_2_, Al_2_O_3_, Fe_2_O_3_, and LiO as major oxides (Table 3). Concentration of these elements varies widely among rock types, so health issue related to water interaction with rocks is different according to the geographic distribution of the rock types.

The chemical transmission of health risk in fact began from rocks with enhanced or reduced contents of elements of greater significant for life (Davis 1966). Health issue related to water and stream sediments in the study area is generally derived from the basement rocks upon weathering. Stream waters drained through soil waste disposals from artisanal gold panning sites, which could contain high level of contaminants, are supposed to be as a secondary source of water pollution in the area. Agricultural fertilizers and pesticides released into the soil might be also considered as important factors in concentration of some trace elements in surface and groundwater of the area.

### Hydrogechemistry

The chemical composition of rocks, mineral, and soils through which the groundwater flows causes very large variations in the chemistry of the groundwater (Davis 1966). The ground and surface water in the study area are directly using as drinking water, and obviously there is a link between water chemistry and health. The results of elements above MAC and elements which have health impact in its low level such as selenium are presented and discussed as follows.

*Bromine* Maximum acceptable concentration (MAC) of 0.01 mg/l for bromine in drinking water has been established on the basis of health consideration. All the results are found to be above WHO maximum allowable level—0.01 mg/l (Table 2). The values of Br could be related to the use of fertilizer and pesticides in agriculture and artisanal gold mining practice.

The bromine concentration in rocks, soils, and freshwater usually is very small. The values of Br could be related to the fertilize and pestsides uses in agriculture and artisanal gold mining practice.

Some of health effects that can be caused by excess bromine value in water are malfunction of nervous system, gastrointestinal, and disturbances in genetic material and also cause damage to organs such as liver, kidneys, lungs, and thyroid glands. Some forms of organic bromine, such as ethylene bromine, can even cause cancer (US EPA 2001).

*Fluorine* The World Health Organization (WHO) guideline value for fluorine in drinking water is 1.5 mg/l, but in most samples about (55.5%) of the fluoride contents were above the 0.5 mg/l, the limit recommended for tropical countries by WHO (Komatina 2001). Some other researchers have been suggested 0.7 mg/l as an action level for tropical countries with high daily intake of water (US EPA 2001; Komatina 2001) and have also been noted the WHO recommended levels of 1.5 mg/l fluorine in drinking waters are not acceptable for hot and dry climate.

Fluoride enrichment in the surface and groundwater could be related to leaching of rocks rich in fluorine such as granite (i.e., as granite sample in the study area constitutes fluorine bearing minerals such as biotite, hornblende, and sphene) and muscovite schist, where this rock unit also contains muscovite, biotite, and feldspar minerals which are considered as fluorine-rich minerals. Rock–water and soil–water interaction, weathering, and leaching of fluorine bearing rocks and minerals could be considered as important factors in the concentration of fluoride in the surface and groundwater.

Excessive intake of fluoride-rich water can cause dental fluorosis (mottling of teeth), skeletal fluorosis (depilating disease, which affects bones), and harm nerves and muscles. The high fluoride is not only possible risk factor but it has some relationship with other liver related causes or it could even increase the severity of the liver related disease (Tigray Health Bureau 2009). Many animal experiments reported that the kidney damage can occur even at low levels of fluoride exposure over large period of time (Liu 2005).

*Aluminum* The study area shows maximum level in groundwater to be 553 ppb. As Al is found in most rocks abundantly, the values could be related to natural sources (i.e., rocks, soils, and their derivative minerals) and to some extent anthropogenic activities such as artisanal gold mining and agriculture. Al in the surface and groundwater could be derived from weathering of rocks rich in Al and leaching of soils accumulated from such activities as artisanal gold mining and agriculture.

Aluminum is included in the priority list of hazardous substance identified by agency for toxic substance and disease register (ATSDR). Sign and symptoms of aluminum toxicity include colic, dementia, esophagitis, gastroenteritis, kidney damage, and liver damage (US EPA 2001).

*Arsenic* The high amount of arsenic value in water of Asgede area could be introduced through anthropogenic activities (i.e., artisanal gold mining) and the dissolution of arsenic containing bedrock (phyllite/slate) and minerals such as pyrite and chalcopyrite.

Ingesting inorganic arsenic contaminated drinking water causes skin cancer, tumors of the bladder, kidney, liver (the primary carcinogen), and lungs. High level of inorganic arsenic in food or water can be fatal, a high level 60 parts per million of food or water (60 ppm) (US EPA 2001). Adverse health effects of too high arsenic value in drinking waters have recently received much attention (Smith 2000).

*Selenium* The selenium concentration in water is generally very low and only rarely exceeds the WHO safely limits of 10 µg/l (Fordyce et al. 2000). The study area shows about 40% of the sample analyzed was < 1 µg/l and only one sample (AT-20) exceeds the WHO safe limits, and this could be related to artisanal gold mining practice in that river.

Concentration of selenium in freshwater around the world is 0.2 µg/L (Wang 1991) and in Asgede Tsimbla area selenium concentration in some well waters observed as low as 0.01 µg/L.

Selenium appears to be essential element in human nutrition. It is part of the biological important enzyme glutathione peroxidase (PHS-PX) which acts as antioxidant preventing tissue degeneration. Selenium prevents the toxicity of several other metals such as silver, mercury, cadmium, and lead (US EPA 2001). Selenium, at trace levels, is essential in human and animal diet, and its deficiency has received much attention. It causes symptoms such as muscular degeneration, impeded growth, fertility disorders, anemia, and liver disease (Wang 1991). Keshan and Kaschin–Beck diseases reported on regional scale from China are caused by Se deficiency. The recommended daily intake described by many researchers and agencies for Se is about 35 µg per day, and doses larger than 200 µg can be toxic (Lag 1984). Other researchers, e.g., Fordyce et al. (2000), FDA (1998), and Galagan and Lamson (1953) had noticed deficiency level (< 11 µg/g per day) and toxic level (> 900 µg/g per day) for selenium. Anyway Se is an element that should better have a minimum guideline level by WHO.

Iron and magnesium fall above MAC, but there is no evidence of adverse health effects specifically attribute to these elements in drinking water; rather they have more aesthetic nature (cause undesirable test and odor to the water) or limit the use of water for practical purpose.

#### Bivariate correlations

Cu and Se show similarity in distribution pattern and have positive correlation. The trend between Cu and Se could origin from dissolution of sulfide minerals. The similar behavior of Cu and Se can be explained by their geochemical similarity, which are chalcophile elements (prefer to bond with sulfur). The trend between Al and Fe also indicates strong positive correlation. This could be interpreted as indicative of common source for them. Fluorine shows negative correlation with bromine, and this could indicate that they have different origins.

### Stream sediment geochemistry

The accumulation of heavy metals in sediments can be a secondary source of water pollution, once environmental condition is changed (Cheung et al. 2003). There for an assessment of heavy metal contamination in sediment is an important tool to assess the risk of hydrogeochemical environment. Assessments of metal contaminants in the area are discussed below.

#### Assessment according to United State Environmental Protection Agency (US EPA)

The chemical contamination in sediments was evaluating by comparison with sediment quality guideline proposed by US EPA (2001). These criteria are shown in Table 4. Pb in all stations under investigation was belong to unpolluted sediments, while elements Cu, Ni, and Mn are considered as moderately polluted, and Cr more or less belong to heavily polluted.

**Table 4:** US EPA guidelines for sediment quality as compared to present study—Shire area

Metal	Not polluted	Moderately polluted	Heavily polluted	Present study
Ag	–	–	–	1.1–5.3
Cu	< 25	25–50	> 50	11–75
Ni	< 20	20–50	> 50	18–65
Pb	< 40	40–60	> 60	1.1–5.3
Co	–	–	–	18–54
Cr	< 25	25–75	> 75	68.9–251
Mn	< 300	300–500	> 500	229–1100

## Conclusion

The ground and surface water in some zones of the study area have values of Br, F, Al, As, Pb, U, Fe, Mg, and NO_3_ exceeding MAC guideline standards for water. Br and F account for almost all the elevated values. The association of F, Br, and Cl is found as geochemical indicator of liver cancer in areas such as Parana–Brazil, China and Sri Lanka (Islam et al. 2000).

Trace elements like Cd, Cr, Pb, Be, and Tm which have serious health effects fall to pass quality standards set for drinking water. Only two sample sites fall above MAC for As, and these may not considered as a major problem in the area. From the present study, the water quality is therefore better than expected. The groundwater is generally weak acidic to basic and the dominate ions are Mg and NO_3_.

It is clear that health relates not only to excesses of trace elements in drinking water supplies, but may also relate to deficiencies (e.g., Se). Most water and stream sediments analyzed carried out in the study area shown significantly low as compared to worldwide values of Se for these geochemical media.

The accumulation of heavy metals in sediments can be a secondary source of water pollution, once environmental condition is changed (Cheung et al. 2003). The geoaccumulation index (Igeo) of some heavy metals such as chromium, cobalt, copper, and lead is calculated for stream sediment quality in the study area and gave values from unpolluted to moderately polluted.

Although liver-related disease has been identified around Shire area, the causes of the disease remain unclear. Environmental factors are mostly considered to explain the etiology of this liver-related disease.

## Figures and Tables

**Table 8:**
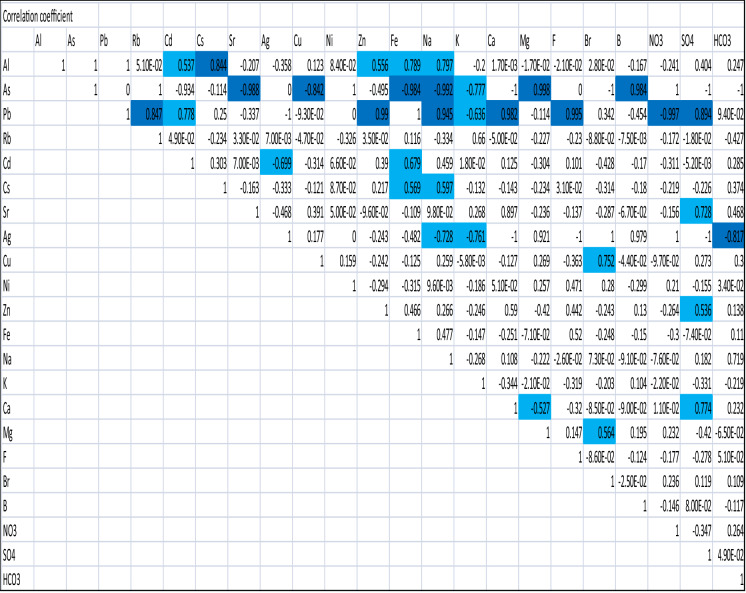
Correlation matrix, all wells

Correlation stronger than 0.5 is lightly marked and correlation stronger than 0.8 is darkly marked

## Appendix 1

See Table 5.

**Table 5:** Descriptions of water (ground and surface) sampling sites

No	Location	Date (2010)	Easting (UTM)	Northing (UTM)	Altitude (m)	Remarks
AT-01	Mai Weyle stream	10 March	374,261	1,558,110	1015	Surface water
AT-02	Mai Weyle	10 March	374,469	1,558,419	1021	Groundwater
AT-03	Mai Hanse	10 March	376,595	1,560,655	1123	≫
AT-04	Mai Lomin	11 March	379,046	1,561,664	1133	≫ (Hand-dug well)
AT-05	Mai Lomin stream	11 March	374,188	1,560,335	1028	Surface water
AT-06	Mai Hammar	12 March	375,764	1,561,115	1031	Groundwater
AT-07	Mai Hammar	12 March	378,681	1,561,598	1043	Groundwater
AT-08	Mai Hammar	12 March	376,232	1,562,233	1068	Surface water
AT-09	Mekayha	13 March	377,713	1,557,743	930	Surface water (Pond)
AT-10	Mekayha	13 March	377,671	1,558,074	998	Groundwater
AT-11	Fesfesay stream	14 March	377,594	1,560,196	1011	Surface water
AT-12	Fesfesay	15 March	377,676	1,560,049	1022	Groundwater
AT-13	Fesfesay	16 March	380,122	1,557,807	1066	≫
AT-14	Mai Teli	17 March	379,171	1,560,392	968	≫
AT-15	Mai Hammar	17 March	380,229	1,566,550	1098	≫
AT-16	Sembel	18 March	379,621	1,563,828	1098	≫
AT-17	Sembel	18 March	379,506	15,633,261	977	≫ (Hand-dug well)
AT-18	Sembel stream	18 March	388,474	1,559,836	1074	Surface water
AT-19	Mai Teli stream	20 March	388,301	1,561,182	1083	Surface water
AT-20	Mai Teli	20 March	379,296	1,561,675	1032	Groundwater

## Appendix 2

See Table 6.

**Table 6:** Trace element chemical analysis of water samples

No	Al (µg/l)	As (µg/l)	Pb (µg/l)	Fe (µg/l)	Cd (µg/l)	Tm (µg/l)	Ni (µg/l)	Co (µg/l)	Se (µg/l)	U (µg/l)	B (µg/l)
1	< 0.01	< 0.01	< 0.01	10	0.087	0.021	< 0.01	0.321	0.53	0.066	4.0
2	2.5	2.8	0.21	78 306	0.029	0.023	< 0.01	0.098	2.13	0.106	44.3
3	< 0.01	< 0.01	< 0.01	78	0.158	0.022	0.015	0.245	< 0.01	1.288	7.5
4	2.5	2.8	0.21	< 0.01	0.207	0.021	< 0.01	0.209	0.96	0.702	10.7
5	< 0.01	< 0.01	< 0.01	1939	0.168	0.029	< 0.01	0.255	< 0.01	4.363	10.9
6	< 0.01	< 0.01	< 0.01	370	0.150	0.026	0.097	0.558	< 0.01	0.050	14.7
7	553	< 0.01	24.16	99	0.121	0.026	< 0.01	0.178	< 0.01	1.318	9.0
8	127	< 0.01	< 0.01	16	0.083	0.022	0.025	0.182	4.26	0.173	165.3
9	155	< 0.01	< 0.01	37	0.091	0.021	0.012	0.171	3.36	0.349	117.2
10	36	23.7	< 0.01	383	0.080	0.032	0.255	0.123	4.06	0.633	23.0
11	< 0.01	20.2	< 0.01	< 0.01	0.078	0.021	0.156	0.125	< 0.01	1.321	14.5
12	313	< 0.01	< 0.01	26	0.089	0.021	0.114	0.227	3.34	0.433	18.1
13	< 0.01	< 0.01	< 0.01	197	0.077	0.022	< 0.01	0.237	4.37	0.765	54.2
14	3	< 0.01	< 0.01	20	0.087	0.027	< 0.01	0.273	3.48	1.885	< 0.01
15	113	< 0.01	< 0.01	990	0.105	0.025	0.003	0.160	0.21	0.071	17.9
16	< 0.01	< 0.01	< 0.01	77	0.073	0.023	< 0.01	0.406	4.60	0.144	55.3
17	42	< 0.01	< 0.01	27	0.092	0.020	< 0.01	0.178	4.70	1.056	50.7
18	46	< 0.01	< 0.01	5	0.068	0.020	0.027	0.296	4.70	1.975	< 0.01
19	17	< 0.01	< 0.07		0.101	0.021	0.011	0.249	6.85	0.507	19.0
20	< 0.01	< 0.01	< 0.01		0.101	0.021	< 0.01	0.249	13.99	0.507	19.0

No	Ba (µg/l)	Be (µg/l)	Ce (µg/l)	Cs (µg/l)	Sm (µg/l)	Rb (µg/l)	Sr (µg/l)	Ag (µg/l)	Cu (µg/l)	Zn (µg/l)
1	44	0.051	0.034	0.27	0.026	0.89	4338	< 0.01	0.41	13.53
2	23	0.027	0.033	0.21	0.027	0.85	115	< 0.01	0.85	4.84
3	167	0.031	0.192	1.60	0.050	1.06	1434	< 0.01	0.21	1.31
4	68	0.024	0.029	2.60	0.022	0.17	1597	< 0.01	0.36	11.78
5	67	0.076	1.660	1.93	0.212	1.23	1488	0.27	0.97	11.94
6	56	0.044	0.274	1.32	0.060	2.23	292	< 0.01	0.22	5.33
7	83	0.043	0.742	1.16	0.107	0.30	524	< 0.01	0.69	1.24
8	118	0.027	0.245	0.87	0.046	4.86	302	< 0.01	0.96	2.88
9	68	0.022	0.068	0.60	0.030	1.26	307	< 0.01	0.54	0.24
10	14	0.039	1.953	0.54	0.295	0.49	87	< 0.01	1.70	0.52
11	59	0.023	0.010	2.80	0.023	0.59	420	< 0.01	0.66	< 0.01
12	97	0.024	0.123	1.03	0.039	1.23	527	< 0.01	1.55	< 0.01
13	82	0.027	0.148	1.06	0.047	1.11	503	1.69	1.35	6.10
14	77	0.026	0.079	1.48	0.043	0.88	716	< 0.01	0.99	< 0.01
15	10	0.026	0.120	1.23	0.040	0.52	178	< 0.01	0.07	< 0.01
16	73	0.027	0.261	0.82	0.050	0.92	444	< 0.01	0.75	6.03
17	40	0.021	0.064	0.85	0.029	0.68	601	< 0.01	0.86	1.77
18	81	0.020	0.051	1.59	0.028	0.55	843	< 0.01	0.65	< 0.01
19	105	0.021	0.206	0.80	0.049	0.97	703	0.35	1.58	3.39
20	81	0.021	0.206	0.80	0.043	0.49	87	0.30	1.35	0.52

## Appendix 3

See Table 7.

**Table 7:** Major ions chemical analysis and water type

Sample no	F	Br	NO_3_	SO_4_	HCO_3_	K	Na	Ca	Mg	Water type
AT-01	0.3737	0.4743	0.3595	435.3391	299.8	1.3154	23.1404	252.8269	7.5592	Ca–SO_4_–Cl–HNO_3_
AT-03	0.01	0.883	51.3786	13.3591	278.75	3.9794	7.5569	29.6082	93.5829	Mg–HNO_3_–Cl
AT-05	0.6964	0.1067	24.5498	22.7953	872.00	2.3244	77.1731	112.5096	30.9147	Sr–Na–HCO_3_
AT-06	1.0424	0.2695	8.8919	46.0297	616.00	2.664	128.897	90.3463	47.5582	Ca–Na–Mg–HCO_3_
AT-07	3.5244	0.4623	0.198	182.4118	740.18	3.5602	166.7217	60.4901	41.0573	Na–HCO_3_–Cl–SO_4_
AT-08	0.2506	0.2031	0.0069	58.8343	538.34	8.3995	24.2788	36.4384	16.9719	Ca–Mg–Na–HCO_3_–Cl–SO_4_
AT-09	0.7904	0.3267	1.0275	150.7578	116.32	3.2922	75.1462	42.7848	20.1805	Na–Ca–HCO_3_–Cl–SO_4_
AT-10	0.01	0.9031	34.5338	16.3117	191.96	4.7115	13.3544	36.8005	126.7885	Mg–Ca
AT-11	0.3396	0.9303	18.7139	21.5874	0.17	5.5722	35.1257	45.7797	103.3873	Mg–Ca–Na
AT-12	0.01	1.3663	0.5617	159.1126	55.63	1.4776	112.6191	8.8124	87.104	Mg–Na–HCO_3_–Cl
AT-13	0.747	0.2089	1.6022	45.8517	718.71	4.0057	49.7108	39.4576	28.872	Mg–Na–Ca–Cl–HCO_3_
AT-14	0.7309	0.9971	0.4488	226.5273	260.06	3.2033	78.7514	97.5354	54.7073	Ca–Na–SO_4_–HCO_3_–Cl
AT-15	0.4872	1.4755	2.4962	116.0967	263.07	2.2248	60.328	58.4001	75.0533	Mg–Ca–Na–HCO_3_–Cl–SO_4_
AT-16	1.2021	0.941	14.6134	145.5691	261.66	5.984	108.8469	106.7415	53.5304	Ca–Na–Mg–HCO_3_–Cl
AT-17	16.4932	0.506	0.2541	1.1178	681.86	1.5381	47.1395	11.0219	68.832	Mg–Cl–HCO_3_
AT-18	0.3493	0.6147	1.6146	105.3168	335.43	4.5565	52.6247	43.4327	91.0991	Mg–Cl–HCO_3_
AT-19	0.3512	0.5082	1.7603	104.8366	316.93	4.5399	52.5605	43.365	91.0324	Mg–Na–Cl
AT-20	0.6599	1.0503	51.752	140.3853	648.16	1.8192	134.5995	110.9888	36.0528	Na–Ca–Cl–HCO_3_

## Appendix 4

See Table 8.

## Appendix 5

See Table 9.

**Table 9:** Occurrence of some diseases and their possible contaminants

Parameters	MAC (µg/l), WHO, EU and Canadian	Potential health effects		Source of contaminants
		Deficiency	Abundance	
As	10	Insufficient hair growth Spleen enlargement	Skin cancer Tumors of bladder, kidney, liver, and lungs	Natural deposit, electronic waste
Pb	10		Kidney, liver, heart and nervous system damage	Natural and industrial deposits
Se	10	Breast, gastrointestinal and skin cancer Liver damage and heart muscle disorder	Hair and nail loss Nervous disorder	Natural deposit, mining, smelting, and coal combustion
Tm	10		Kidney, liver, brain, and intestinal effects	Electronic, drug, and alloy
Br	10		Malfunctioning of nervous system Liver, kidney, lung and thyroid gland damage	Natural deposit
Cr	50	Diabetes	Liver, kidney and circulatory system disorders Lung cancer	Natural deposit, mining, and electroplating
Cu	1000	Anemia	Gastrointestinal irritation	Natural/industrial deposit, wood preservation
Al	100		Kidney and liver damage Dementia, colic, and esophagitis	Natural/industrial deposits
F	1500	Developmental defects of bone and teeth	Dental and skeletal fluorosis	Natural deposits
NO_3_	45,000		Methemoglobinemia	Animal waste, fertilizer, and natural deposit
Cd	10	Growth reduction	Kidney effects Cancer of prostate	Natural deposit Galvanized pipe corrosion
Mg	50,000	Connection with cancer pathogenesis	Anestesis Connection with cardiovascular disease (Ca + Mg)	Natural deposit

Selected parameters in geochemical environment (surface and groundwater) on the occurrence of some diseases and their possible source of contaminants

## Appendix 6

See Table 10.

**Table 10:** Modal composition (vol%) and major petrographic characteristic of rocks

Rock type	Petrographic description
Feldspar–Sericite schist Sri (39), Fs (22), Ep (16), Chl (15), Qtz (5) Acc (3)	Gray with pale greenish yellow tint in color and fine grained in texture. Discontinuous like veins of quartz, feldspar, and opaque minerals is observed along the thin section
Quartz–Biotite schist Bt (32), Qtz (30), St (15) Fs (10), Grt (6), Chl (2)	Gray in color and fine grained in texture; large crystals of staurolite and garnet are seen as porphyroblasts over a schistose matrix. Veins of quartz are observed along the section
Quartz–Graphite schist Gr (50), Qtz (30), Bt (15), Fs (5)	Dark gray in color and fine grained in texture; intensive recrystallized and lens like veins of quartz are seen on the schistose matrix. Feldspar is altered to sericite
Chlorite–Talc schist Tic (42), Chl (32), Cal (170), Fs (5), Qtz (2), Acc (2)	Light gray with faint greenish tint in color and fine grained in texture. Matrix is mainly composed of platy talc, chlorite, and xenoblastic calcite which have strong parallel alignment
Graphite–muscovite Schist Ms (25), Gr (20), Qtz (18), Bt (13), Sri (12), Fs (10)	Gray in color and fine grained in texture; veins field by feldspar and quartz is crossing in the matrix. Biotite is replaced by muscovite and quartz is recrystallized
Biotite–Hornblende–Granite K-fs (23), Pl (20), Qtz (15), Hbl (12), Bt (10), Ep (7), Spn (5), Chl (3), Acc (3)	Pink and white with black spots in color and very coarse to medium grained in texture; k-feldspar is sericitized and hornblende is replaced by chlorite

Mineral abbreviations are according to Kretz (1983). *Qtz* quartz, *Bt* biotite, *Ms* muscovite, *Gr* graphite, *Tic* talc, *Chl* chlorite, *Sri* sericite, *Fs* feldspar, *Hbl* hornblende, and *Acc* accessories
